# Non-random mating behaviour between diverging littoral and pelagic three-spined sticklebacks in an invasive population from Upper Lake Constance

**DOI:** 10.1098/rsos.241252

**Published:** 2025-01-15

**Authors:** Tobias Zeidler, Albert Ros, Samuel Roch, Arne Jacobs, Juergen Geist, Alexander Brinker

**Affiliations:** ^1^ Fisheries Research Station Baden-Württemberg, Argenweg 50/1, 88085 Langenargen, Germany; ^2^ School of Biodiversity, One Health, and Veterinary Medicine, College of Medical, Veterinary & Life Sciences, University of Glasgow, Glasgow G12 8QQ, UK; ^3^ Department of Life Science Systems, Aquatic Systems Biology Unit, Technical University of Munich, TUM School of Life Sciences, Mühlenweg 22, 85354 Freising, Germany; ^4^ University of Constance, Institute for Limnology, Mainaustraße 252, 78464 Konstanz, Germany

**Keywords:** reproductive isolation, mate choice, aggression, courtship, adaptive divergence, adaptive radiation

## Abstract

Adaptive divergence and increased genetic differentiation among populations can lead to reproductive isolation. In Lake Constance, Germany, a population of invasive three-spined stickleback (*Gasterosteus aculeatus*) is currently diverging into littoral and pelagic ecotypes, which both nest in the littoral zone. We hypothesized that assortative mating behaviour contributes to reproductive isolation between these ecotypes and performed a behavioural experiment in which females could choose between two nest-guarding males. Behaviour was recorded, and data on traits relevant to mate choice were collected. Both females of the same and different ecotypes were courted with equal vigour. However, there was a significant interaction effect of male and female ecotypes on the level of aggression in females. Littoral females were more aggressive towards pelagic males, and pelagic females were more aggressive towards littoral males. This indicates rejection of males of different ecotypes in spite of the fact that littoral males were larger, more intensely red-coloured and more aggressive than the pelagic males—all mating traits female sticklebacks generally select for. This study documents the emergence of behavioural barriers during early divergence in an invasive and rapidly diversifying stickleback population and discusses their putative role in facilitating reproductive isolation and adaptive radiation within this species.

## 1. Introduction

A key feature of speciation is reproductive isolation, which limits gene flow between populations [1] and facilitates the build-up of genetic differences [2]. Reproductive isolation involves pre- and post-zygotic barriers [3]. Pre-zygotic barriers encompass all those that impair mating between incipient species, including selective mate choice as well as spatial and temporal separation during reproduction [4,5], sperm competition [6] and cryptic female choice [7]. Post-zygotic barriers manifest after mating and result from fitness disadvantages of hybrid traits [8–10]. Since Darwin’s seminal theories on the origin of species [11], research has focused on the way reproductive isolation can arise [2,12–15], and a consensus has emerged that natural selection on the viability of individuals during adaptation to alternative environments can be an important driver [16]. Sexual selection is driven by competition for access to gametes and fertilization [17–19], which may result in a genetic linkage between secondary mating characters and mate choice through divergent runaway processes [20–23]. In general, reproductive isolation will most likely evolve when processes of adaptation to alternative environments are reinforced by selective mate choice [19,24–27]. Studying micro-evolutionary processes at an early stage of speciation, especially in sympatric populations, is thus essential in understanding how adaptive divergence and selective mate choice act together to initiate the evolution of reproductive isolation [28].

The three-spined stickleback species complex (*Gasterosteus aculeatus*, Linnaeus, 1758; hereafter referred to as stickleback) contains many evolutionarily recent species pairs, making it an ideal system for studying micro-evolutionary processes [29]. Sticklebacks are widely distributed in boreal and temperate regions of the northern hemisphere and have a marine origin [30]. The ancestral marine form repeatedly colonized inland habitats after the Last Glacial Maximum [31], and freshwater ecotypes have evolved subsequently and in parallel by adaptation from standing genetic variation [32–35]. Different ecotypes exist, which are locally adapted, and incipient species pairs have evolved along environmental gradients, for instance, ocean–freshwater [36,37], stream–lake [38–40] and benthic–limnetic [41,42]. Benthic–limnetic stickleback species pairs have exclusively been described in seven lakes, all in the Strait of Georgia region of British Columbia [29,41–44]. In these lakes, the pelagic ecotype always has a small, fusiform body and fine gill rakers to increase plankton foraging efficiency. In contrast, the benthic ecotype has a large, deep body and a higher suction force as an adaptation to feeding on benthic prey [45–51]. Although many stickleback ecotypes have evolved independently at different locations, ecological adaptation has often led to similar phenotypes in similar environments [35,52,53]. The repeated evolution of stickleback ecotypes represents an ideal system to study contemporary evolution and provides strong evidence for the role of ecology in speciation [37,54].

Divergence into ecotype pairs can be facilitated by assortative mating on phenotypic characters that correlate with the ecotypes [4,39,55–60]. This would likely be based on common sexual selection processes in the stickleback in which mates are selected based on visual appearance [61,62], courtship behaviour [36,63], nest structure [64], sexual imprinting [65] and chemical cues [66,67]. Females typically prefer intensely red-coloured males [68,69]. Size has also been shown to play an important role in mate choice since many ecotype pairs show assortative mating on size, and size is often divergent between environments [37,54]. An important driver of the formation of ecotype pairs would be when individuals of a specific ecotype develop a preference for characters that signal local adaptation to their respective environments [70], and such processes might contribute to the rapid evolution, within a few generations, of stickleback species pairs in oceanic–freshwater sticklebacks [35,56,71].

In Lake Constance, Germany, an invasive population of three-spined sticklebacks shows early signs of divergence between littoral and pelagic individuals [72]. Repeated anthropogenic introductions from the end of the nineteenth century eventually led the species to invade the littoral zone of the lake, where it was well established by 1962 [73–76]. In 2012, sticklebacks from this littoral population invaded the pelagic zone within one season [75,77] in such large numbers that they accounted for more than 95% of fish abundance and 28% of biomass in this habitat by 2014 [78]. However, as sticklebacks depend on breeding in nests that are built and defended by males [79], this pelagic population still returns to spawning grounds in shallow areas in late spring to early summer, where it breeds sympatrically with littoral sticklebacks [80]. Despite an expected high rate of gene flow, low genome-wide genetic differentiation between populations and their shared recent history, these littoral and pelagic sticklebacks exhibit several regions of localized differentiation across the genome [72]. The ecotypes differ significantly in body shape, with littoral sticklebacks being larger and having slightly longer snouts and deeper bodies [72]. These early signs of morphometric and genetic divergence between ecotypes suggest the potential emergence of reproductive isolation and a putative role of mating pattern divergence in the Lake Constance population.

This study investigates whether female sticklebacks show a mating preference when presented with a choice between males of a littoral and a pelagic ecotype, both of which guard nests and attempt to court them. To test this, we conducted a behavioural experiment under controlled laboratory conditions. Because the two ecotypes are in the process of divergence [72], we expected to find evidence for assortative mating behaviour, which relies on divergent traits between the ecotypes. We hypothesized that littoral stickleback females would prefer to mate with littoral stickleback males, and that pelagic females likewise would prefer to mate with pelagic males.

## 2. Material and methods

### 2.1. Fish sampling

**Figure 1.**
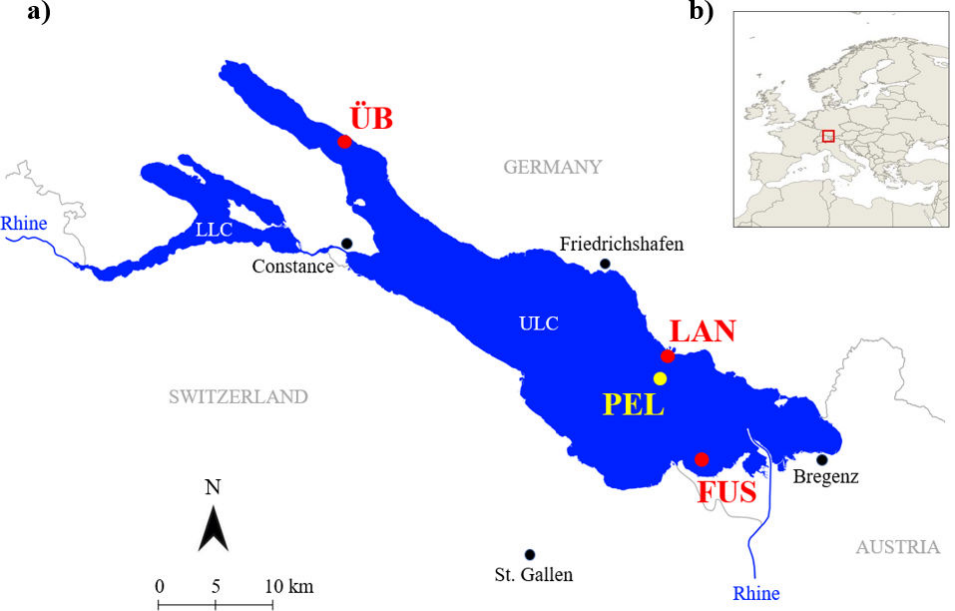
(*a*) Sampling sites in Lake Constance and (*b*) location of Lake Constance in Europe. The sampling sites are abbreviated as ÜB: Überlingen (littoral zone); LAN: Langenargen (littoral zone); FUS: Fussacher Bucht (littoral zone); PEL: pelagic zone. ULC and LLC refer to Upper Lake Constance and Lower Lake Constance, respectively.

Lake Constance is a large oligotrophic pre-alpine lake located in the Rhine drainage basin with shorelines on the borders of Germany, Austria and Switzerland (figure 1). With a surface area of 535 km^2^ and a maximum depth of 253 m, it is the third largest lake in Europe [81]. The body of the lake is divided into two basins, the deep, warm-monomictic Upper Lake Constance (ULC) and the shallower, dimictic Lower Lake Constance (LLC) [82]. The study focuses solely on the stickleback population in ULC.

Sticklebacks were caught at three sites in the littoral zone and one in the pelagic zone (figure 1) between January and March 2023. The littoral zone sampling sites were located close to the shore at Überlingen (47°45′22.54″ N; 9°10′54.87″ E), Langenargen (47°35′9.04″ N; 9°32′55.80″ E) and Fussacher Bucht (47°29′59.10″ N; 9°35′12.69″ E). Three different sites were sampled to account for potential variation within the littoral population. Pelagic sampling was carried out in the area approximately 3 km offshore from Langenargen (47°34′10.47″ N; 9°32′46.53″ E). We chose this sampling design since we expected higher variation among littoral populations than among pelagic populations, given that habitats are more diverse in the littoral zone [83] and pelagic sticklebacks might perform foraging movements over larger distances, resulting in a higher degree of admixture [47,51]. Fish were caught with benthic and pelagic gillnets (length = 20 m; height = 2 m; mesh width = 8 and 10 mm), which were set overnight at a depth of around 10 m. The sticklebacks were captured in winter, as there is a clear separation between littoral and pelagic individuals during this season. Pelagic sticklebacks migrate inshore to spawn in early spring, where they mix with the littoral conspecifics [80]. Therefore, targeted sampling required capturing the fish prior to the spawning season. Ecotype was assigned by whether the fish was caught in the pelagic or littoral zone. We did not select individuals by outer appearance to keep the experiment unbiased. Dahms *et al*. [72] showed evidence of divergence of littoral and pelagic individuals and our sampling design ensured that the fish participating in the mate choice trials matched the described ecotypes. The fish were kept in eight identical outdoor tanks (77 × 68 × 68 cm^3^) with a continuous water supply from the lake, separated by sampling sites. They were fed ad libitum with frozen bloodworms and artemia two to three times a day until the experiments, which took place in July 2023.

### 2.2. Mate choice experiment

**Figure 2.**
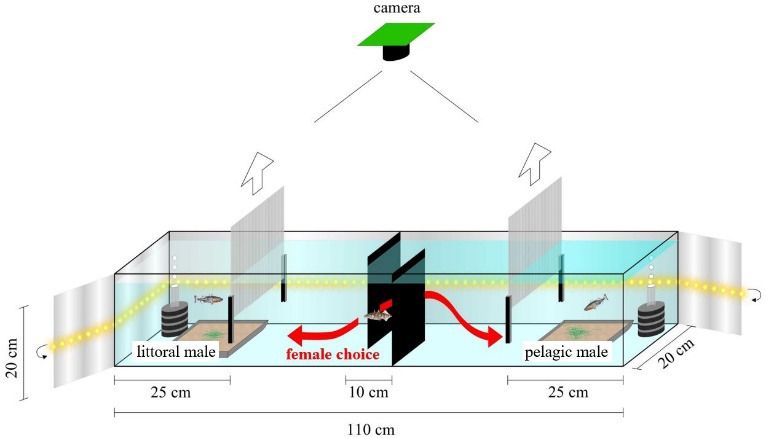
Setup of the aquaria for the mate choice experiment. A milk glass film was wrapped around the aquarium along with an LED strip to optimize illumination for video recording. The male sticklebacks were provided with a tray of sand and green polyester threads to build nests in their respective confined sections in the run up to the trial. Two opaque black separators were mounted in the middle of the aquarium, offset so as to prevent the males from seeing each other. Before each mate choice trial, the stimulus females and the transparent separations were removed, and the test female was introduced in the neutral zone between the separators.

In the experiment, females were given a choice between a littoral and a pelagic male, and the mating behaviour of all three fish was recorded. The experiment was conducted in twelve aquaria (110 × 20 × 20 cm^3^; Amazonasbecken.eu, Germany) with identical setups (figure 2). In each aquarium, a littoral and a pelagic female were presented to the males in two subsequent trials and random order. Each female was used in only one trial. In total, 24 females participated in the experiment. Littoral and pelagic males were distributed randomly between the left and right sides of the aquaria. Littoral fish were included in equal numbers from all three sampling sites, and it was accounted for that they were tested against an equal number of mates from the same site to ensure the experiment was balanced (electronic supplementary material, figure S1). The observation time was one hour, and all trials were video recorded with a Raspberry Pi camera (12.3 MP, 7.9 mm diagonal image size; lens: 3.2 mm, 12 MP, 1/1.7″), mounted 70 cm above the bottom of the aquaria and connected to a Raspberry Pi computer. Side illumination through an LED strip (Q. Laomi, China) and a white bottom surface were used to improve the visibility of the fish. The behaviour of the fish and the movement of the female were logged using the behavioural observation software BORIS (v. 8.18.1 [84]). An ethogram with 28 behaviours was defined based on the literature (for details, see table 1), and the frequency of these behaviours was counted. The aquarium had male compartments at either end, confined by transparent removable panels (figure 2). These transparent panels were punctured with holes to allow the exchange of water and odours. Additionally, two opaque black partial separators were installed in the middle of the aquarium to prevent the males from seeing each other. This neutral space in the middle served for the introduction of the female at the beginning of each trial. After 30 min of acclimation time, the transparent panels of the male compartments were opened, and the observation began. The areas left and right of the opaque separators in the middle were defined as littoral or pelagic male sides based on the male in residence at either end. For analysis of female time budget, the time spent on each side was measured. All males were stocked in the aquaria on the same day. The experimental trial was then performed after 4 days up to two weeks to give the males enough time to establish territories and build nests. Variation in time was due to some males requiring more time for nest building, but the time was always equal for the littoral and pelagic males in each aquarium. Nest building material comprised a tray (15 × 15 cm^2^) of sand (grain size = 0.4–1.0 mm) and 100 green polyester threads (5 cm). Two females were placed temporarily in the middle of the aquarium in order to stimulate nest-building behaviour in the males [97], and these females were removed prior to the mate choice trials. The nests were examined for completeness. Nests with a well-defined structure and visible entrance were considered complete [98].

**Table 1.** Ethogram with stickleback behaviours. The behaviours were assigned into five categories as stated on the left. Column ‘subj.’ indicates whether the behaviour was exhibited by males (m) or females (f), and column ‘mod.’ denotes whether the behaviour was recorded along with a modifier specifying the fish that was the target of the action.

	behaviour	description	subj.	mod.	reference
overt aggression	bite	quick movement towards another fish, with mouth opening and closing, with physical contact	m, f	yes	[85]
	charge/lunge/attack	fast movement towards another fish, increasing acceleration	m, f	yes	[85]
	chase	one fish attacks and follows while the other fish flees	m, f	yes	[79]
fear	flee	accelerating movement away from another fish or stimulus; constantly swimming against the wall	m, f	no	[85]
	freezing	immobile near the bottom or near the surface of the aquarium	m, f	no	[85]
	show belly spine	pelvic spine erected and pointing towards another fish	m, f	no	[86]
nest-directed behaviour	build	pushing nesting material into position, changing nest structure, removing nesting material	m	no	[87]
	collect	gathering of nesting material and transport to the nest	m	no	[87]
	dig	biting or digging at the substratum, rearrangement of sediment	m	no	[87]
	fan	fanning the nest with pectoral fins	m	no	[88]
	glue	pressing the cloacal opening against the nest and gliding forward while assuming an upward-angled posture	m	no	[87]
	return	male swims back to his own nest	m	no	[89]
courtship behaviour	dorsal pricking	male jerkily pushes the female towards the water surface with the dorsal surface	m	no	[90]
	in nest	fish creeps through the nest	m	no	[79]
	lead–follow	male leads the female to the nest; female follows leading male to the nest	m	no	[79]
	show nest entrance	male points towards the nest entrance	m	no	[79]
	tremble	male gives the female’s rump several prods with a trembling motion after it has entered the nest	m	no	[79]
	zigzag dance	swift series of sideways jumps towards the female	m	no	[88]
	at nest entrance	female inspects nest entrance, or enters with the head only	f	no	[91]
	head-up posture	head is turned upwards; female displays belly full of eggs	f	yes	[92]
	push^a^	fish nudges the other fish with the snout, no biting	m, f	yes	[93]
agonistic behaviour	approach	movement towards another fish	m, f	yes	[85]
	circling	fish circle around each other	m, f	yes	[85]
	frontal display	standing while orienting to the face of the other fish	m, f	no	[85]
	jolt	sudden, jerky movement after being bitten or after physical contact; whole-body shudder	m, f	no	[94]
	lateral display	standing while orienting side-on to the other fish, holding position laterally	m, f	no	[95]
	pendulum	fish moves back and forth several times; in males this often happens at the edge of the territory	m, f	no	[96]
	tail beat	side-to-side sweeping of the tail; sometimes touching the other fish with the tail in a beating motion	m	no	[85]

^a^ The behaviour ‘push’ was considered as a courtship behaviour in females and as an agonistic behaviour in males.

### 2.3. Morphometric and colourimetric measurements

**Figure 3.**
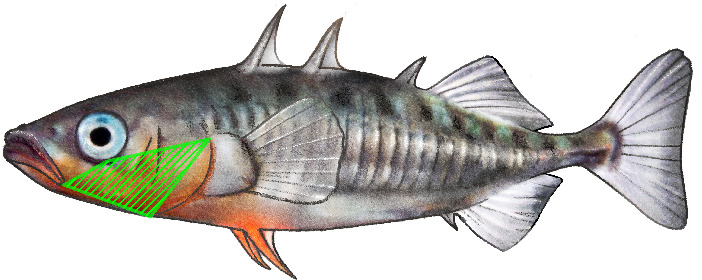
Assessment of male nuptial colouration. The green area defines the polygon from which mean RGB values were obtained. The polygon spans from the end of the mouth opening to the eye and the operculum.

Each specimen was weighed to the nearest 0.1 g and photographed with a Pentax K-3 II digital camera (Ricoh Company Ltd, Japan). The condition of the fish was measured with Fulton’s condition factor, calculated with the formula K = weight/length^3^ [99]. Standard length of the fish was derived from the photos using ImageJ (v. 1.54d) [100]. Male nuptial colouration was measured as described by Berner *et al*. [39]. In the first step, mean RGB values (representing red, green, and blue colour channels) of male nuptial colouration were obtained from a defined area on the cheek (figure 3) with ImageJ. Hue (dominant wavelength) was then derived from the RGB values using the *rgb2hsv* function of the *GrDevices* package, and relative luminance (perceived brightness) was calculated using the formula 0.2126*R + 0.7152*G + 0.0722*B [39].

### 2.4. Data preparation

The behavioural data were arranged with two sets of entries for each female individual, one listing the frequency of all behaviours displayed in the context of the littoral male and another in the context of the pelagic male. Some behaviours were recorded with a modifier to specify the subject and object of the action (e.g. subject = female, behaviour = bite, modifier = right male; see table 1). Behaviours without modifiers were assigned to the male on whose side of the aquarium the female was positioned at that moment. Behaviours were grouped into higher-level categories, as indicated in table 1, by summation of the count data. The count data were normalized for the time the female spent on each respective male’s side by dividing each count by this time. Data from one female that spawned during the experimental trial were excluded from all behavioural analyses since they were not comparable with those of other subjects.

### 2.5. Statistical analysis

#### 2.5.1. Phenotypic characterization

A linear model (LM) was used to test for differences in standard length, weight, condition and male nuptial colouration of littoral and pelagic sticklebacks. In this analysis, weight was log-transformed. The data were tested for normality with a Lilliefors test [101] in the package *nortest* [102]. If the data did not fulfil the criteria for parametric testing, a paired Wilcoxon rank sum test [103] was used. To test the association between nest completeness and male ecotype, the number of complete and incomplete nests was compared between male ecotypes using Fisher’s exact test [104].

#### 2.5.2. Female time budget

A generalized linear mixed-effects model (GLMM) with Gaussian error distribution and a square root link function was used to analyse how female origin affected the time spent on the side of the littoral and pelagic male. This model included time on the respective male side as a dependent variable and male and female ecotypes as explanatory variables (electronic supplementary material, table S1). Estimated marginal means were obtained from the model using the *emmeans* package [105]. As two dependent time measures were analysed per female, female individuals were included as a random factor in this model.

#### 2.5.3. Ecotype effects

Linear mixed-effects models (LMMs) were used to test the effect of male and female origin on the frequency of specific behaviours. In these models, a specific male or female behaviour was the dependent variable, and male and female ecotypes were used as explanatory variables. If the interaction of male and female ecotype was significant, it was inferred that the identity of the mating partner had an effect on the behaviour of the fish. As the behaviour of each female was simultaneously recorded in the context of both a littoral and pelagic male, it was necessary to account for the dependency between these two measures, and for that reason, male and female individuals were included as random factors in the models. The marginal effect of mating partner origins on the frequency of behaviours was calculated based on model fits with the *ggpredict* function of the package *ggeffects* [106]. To control for male phenotype, male nuptial colouration (hue) was included in the LMMs in a *post hoc* analysis (presented in table 2). A paired Wilcoxon rank sum test [103] was used to compare the frequency of behaviours displayed towards potential mating partners from the same and opposite ecotype.

**Table 2.** Effect of male and female ecotype (LIT/PEL), and male nuptial colouration (hue) on the frequency of higher-category behaviours. Analysis of variance (ANOVA) tables for LMMs with the formula behavioural category ~ ecotype male*ecotype female + male nuptial colouration. Significant terms are presented in bold font.

behavioural category	ecotype male	ecotype female	ecotype male:ecotype female	male nuptial colouration (hue)
male aggression	***F*_1,16.89_ =7.164** ***p*** **= 0.0160**	*F*_1,11.29_ = 0.575 *p* = 0.464	*F*_1,10.48_ = 3.675 *p* = 0.083	*F*_1,20.24_ = 2.321 *p* = 0.143
male courtship	*F*_1,19.12_ = 0.869 *p* = 0.363	*F*_1,10.37_ = 1.048 *p* = 0.329	*F*_1,10.14_ = 0.001 *p* = 0.976	*F*_1,20.73_ = 3.073 *p* = 0.094
male nest-directed behaviour	*F*_1,20.29_ = 0.944 *p* = 0.343	*F*_1,10.47_ = 0.706 *p* = 0.420	*F*_1,10.47_ = 0.426 *p* = 0.528	*F*_1,20.50_ = 2.574 *p* = 0.124
male agonistic behaviour	*F*_1,19.30_ = 3.197 *p* = 0.089	*F*_1,10.96_ = 0.009 *p* = 0.925	*F*_1,10.96_ = 0.235 *p* = 0.636	*F*_1,20.26_ = 3.517 *p* = 0.075
female aggression	*F*_1,12.65_ = 0.375 *p* = 0.551	*F*_1,14.88_ = 1.081 *p* = 0.315	***F*_1,10.57_ = 15.557** ***p* = 0.0025**	*F*_1,17.42_ = 3.808 *p* = 0.067
female courtship	*F*_1,13.34_ = 0.222 *p* = 0.645	*F*_1,13.05_ = 1.117 *p* = 0.310	*F*_1,10.15_ = 1.466 *p* = 0.254	*F*_1,18.49_ = 1.044 *p* = 0.320
female fear	*F*_1,11.21_ = 0.027 *p* = 0.873	*F*_1,19.27_ = 0.073 *p* = 0.790	*F*_1,11.07_ = 0.0582 *p* = 0.814	*F*_1,14.49_ = 1.706 *p* = 0.212
female agonistic behaviour	*F*_1,13.89_ = 0.243 *p* = 0.630	*F*_1,15.27_ = 4.110 *p* = 0.061	*F*_1,12.86_ = 0.7193 *p* = 0.412	*F*_1,19.37_ = 0.128 *p* = 0.724

#### 2.5.4. Mate characteristics effects

To test the effect of mate characteristics and mate behaviour in inducing aggressive behaviour, linear mixed-effects models (LMMs) were fitted for female subjects (model 1) and male subjects (model 2), and this analysis was performed separately for littoral and pelagic ecotypes:


$$model\;1:\;female\;behaviour∼\beta_{0}+\beta_{1}\;L_{m}+\beta_{2}\;K_{m}+\beta_{3}\;C_{m}+\beta_{4}\;N_{m}+\beta_{5}\;Aggr_{m}+\beta_{6}Agon_{m}+\beta_{7}Court_{m}+\;\beta_{8}\;Nest_{m}\;+\varepsilon_{1}+\varepsilon_{2}$$

$$model\;2:\;male\;behaviour∼\beta_{0}+\beta_{1}\;L_{f}+\beta_{2}\;K_{f}+\beta_{3}\;Aggr_{f}+\beta_{4}\;Agon_{f}\;+\beta_{5}\;Court_{f}+\beta_{6}\;Fear_{f}+\varepsilon_{1}+\varepsilon_{2}$$

where *β*_0_ is the intercept, *β*_1_–*β*_8_ represent the estimated coefficients for the variables, and the error terms ε_1_ and ε_2_ represent among-male and among-female variation, respectively. The following variables were added to the model: L = length, K = Fulton’s condition factor, C = nuptial colouration (hue), *n* = nest quality (1–3), Aggr = aggressive behaviour, Agon = agonistic behaviour, Court = courtship behaviour, Fear = fearful behaviour, Nest = nest-directed behaviour. Variable subscript letters m and f indicate whether the variable describes a male or female trait. Following the principle of parsimony, non-significant variables (*p* < 0.05) were removed stepwise from these models by taking out variables with the highest *p*-value first. The statistical values for each variable were extracted at the time of removal, and the reduced model was always compared to the previous model using the least squares method (using the Wald chi-square test, *car* package) to confirm the legitimacy of removal. The marginal effect of the remaining variables in the minimum adequate model was calculated with the *ggeffect* function of the *ggeffects* package [106]. All mixed-effects models were fitted with the *lme4* package [107], and F-statistics and denominator degrees of freedom were computed with the Kenward–Roger method [108]. Effect sizes (partial eta-squared, *η*²) were calculated based on the sum of squares from the LMM models using the *effectsize* package [109]. Effect sizes can be interpreted as follows: 0.01 ≤ *η*² < 0.06, small; 0.06 ≤ *η*² < 0.14, medium; *η*² ≥ 0.14, large [110]. Behavioural variables were square root transformed in all models. All statistical analyses were conducted using R Statistical Software (v. 4.2.2 [111]) and RStudio (v. 2023.06.0 [112]).


$$model\;2:\;male\;behaviour∼\beta_{0}+\beta_{1}\;L_{f}+\beta_{2}\;K_{f}+\beta_{3}\;Aggr_{f}+\beta_{4}\;Agon_{f}\;+\beta_{5}\;Court_{f}+\beta_{6}\;Fear_{f}+\varepsilon_{1}+\varepsilon_{2}$$

## 3. Results

### 3.1. Phenotypic characterization

**Figure 4.**
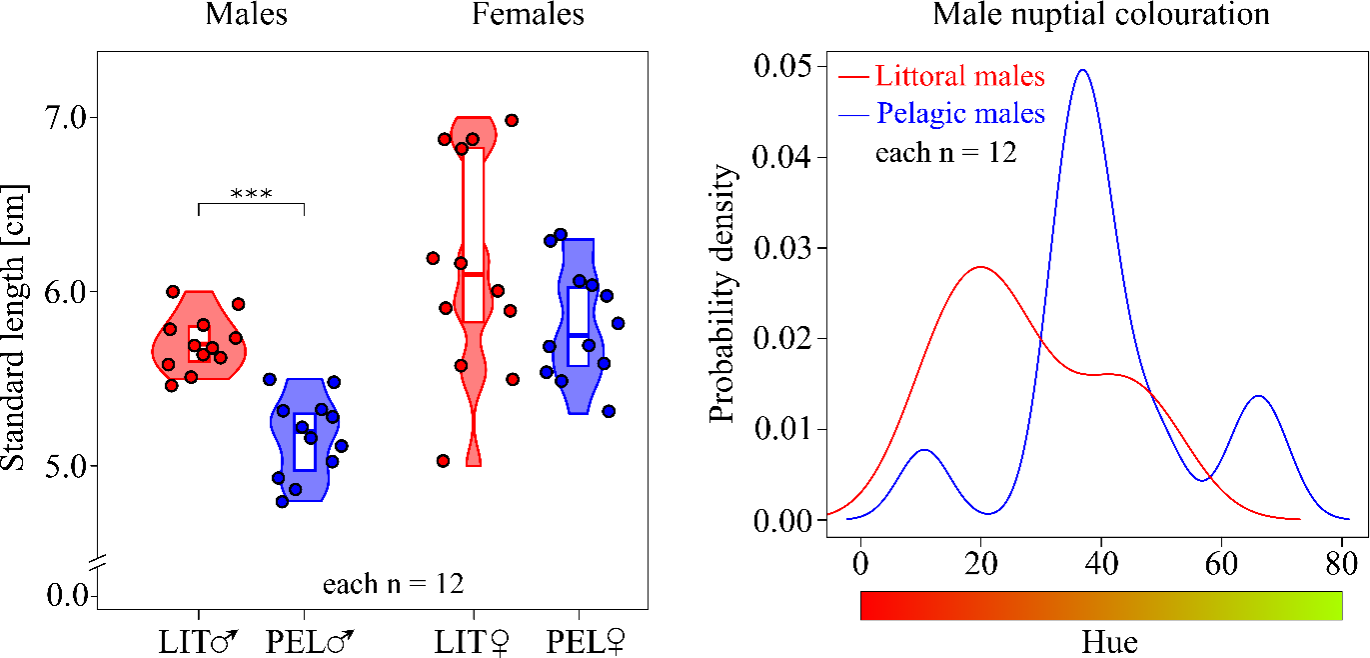
Phenotypic characterization of littoral (red) and pelagic (blue) sticklebacks involved in the mate choice experiment. (*a*) Standard length of males and females visualized by a violin plot showing data distribution, median and interquartile range. Coloured dots represent data points. (*b*) Male nuptial colouration was visualized by a kernel density plot showing the distribution of hue (dominant wavelength) across male individuals. Hue values are given on an angular scale (0–360°), and a colour bar is depicted to facilitate interpretation.

Littoral and pelagic males differed significantly in standard length (LM, *F*_1,22_ = 44.346, *p* < 0.001) and weight (LM, *F*_1,22_ = 23.437, *p* < 0.001), with littoral males being larger than pelagic males (figure 4). Fulton’s condition was not significantly different between both male ecotypes (paired samples Wilcoxon test, *V* = 55, *p* = 0.233). Littoral males displayed a more intense nuptial colouration than pelagic males, as indicated by significantly lower values for both hue (LM, *F*_1,22_ = 4.957, *p* = 0.037) and relative luminance (LM, *F*_1,22_ = 8.613, *p* = 0.008). There was no statistically significant association between ecotype and nest completeness (two-sided Fisher’s exact test, *p* = 0.089). Littoral and pelagic females did not differ significantly in standard length (LM, *F*_1,22_ = 2.739, *p* = 0.112), weight (LM, *F*_1,22_ = 0.969, *p* = 0.336) and Fulton’s condition (LM, *F*_1,22_ = 1.098, *p* = 0.306).

### 3.2. Female time budget

The ecotype of the female test subject had no significant effect on the time spent with the littoral versus pelagic male (non-significant interaction in the GLMM; electronic supplementary material, table S1). Females generally preferred the pelagic male side over the littoral male side (χ^2^ = 5.862, d.f. = 1, *p* = 0.015; electronic supplementary material, table S1). Pelagic females spent on average 27 min on the pelagic male side and 17 min on the littoral male side, while littoral females spent on average 22 min on the littoral male side and 23 min on the pelagic male side (estimated marginal means by the GLMM; see electronic supplementary material, figure S2).

### 3.3. Frequency of higher-category behaviours

**Figure 5.**
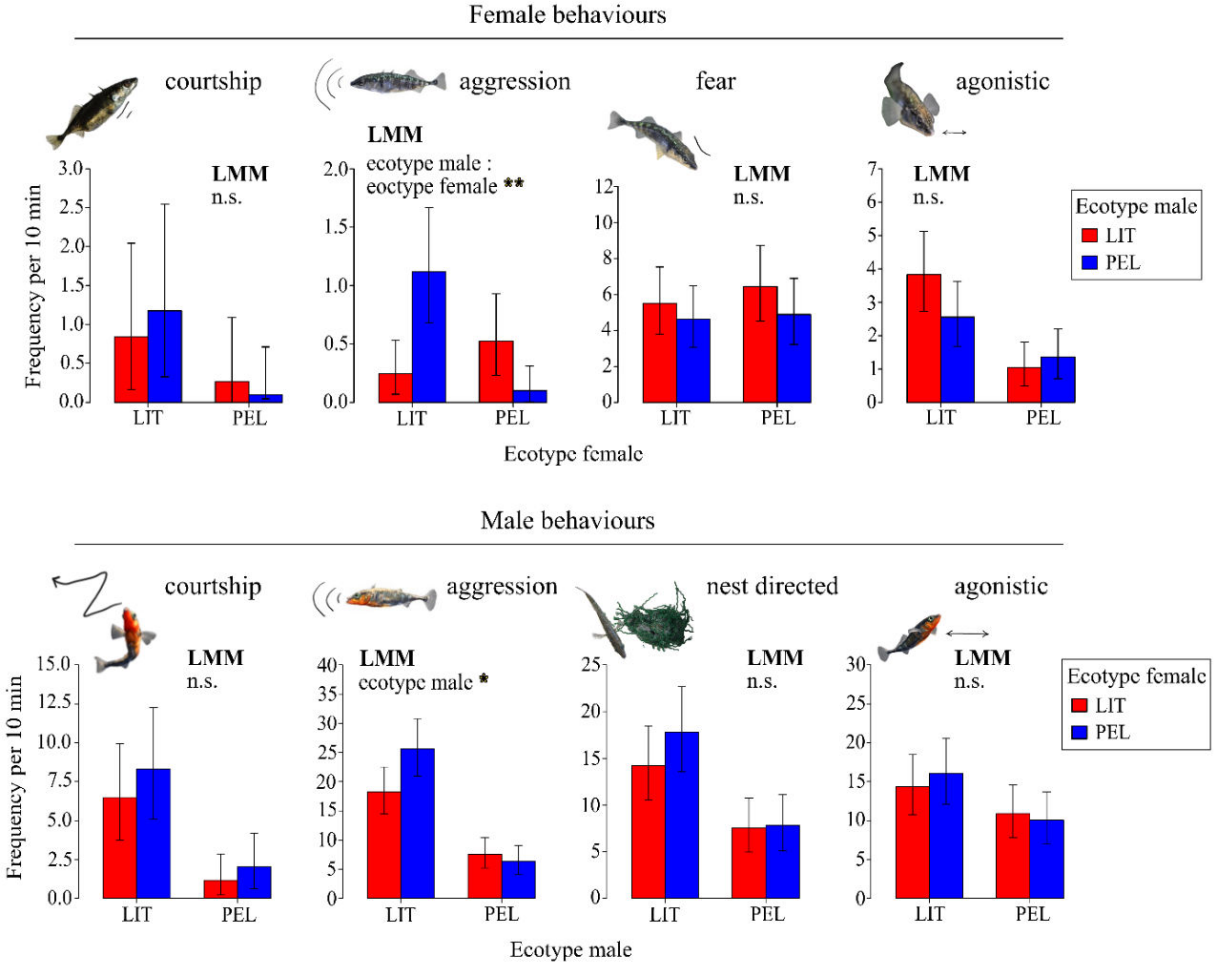
Marginal effect of mating partner origin (LMM) on the frequency of higher-category behaviours, displayed by littoral (LIT) and pelagic (PEL) sticklebacks during encounters with a littoral (red bar) or pelagic (blue bar) potential mating partner. The bar plots show mean and standard errors. Significant effects of male ecotype, female ecotype, and their interaction (ecotype male : ecotype female) are noted above the bar plots. Asterisks indicate significance level: ***p < 0.001; **p < 0.01; *p < 0.05. For more details, see table 2.

The frequency of aggressive behaviour in littoral and pelagic females depended on the ecotype of the male they encountered, as indicated by a significant interaction of male and female ecotypes (LMM, *F*_1,10.57_ = 15.557; *p* = 0.002). Littoral females were more aggressive towards pelagic males, and pelagic females were more aggressive towards littoral males (figure 5). Not distinguishing between female ecotypes, females were generally significantly more aggressive towards the male of the opposite ecotype than towards the male of the same ecotype (paired samples Wilcoxon test, *V* = 103, *p*<0.01). Female courtship behaviour, agonistic behaviour and fearful behaviour were not significantly affected by the male ecotype (table 2). Except for the effect of male ecotype on female aggression, littoral and pelagic females did not generally differ in their behaviour. However, in males, a general behavioural difference was found between the ecotypes. Littoral males were generally more aggressive than pelagic males (LMM, *F*_1,16.89_ = 7.164; *p* = 0.016), and this was independent of the ecotype of the encountered female. No significant differences were found in male courtship, agonistic or nest-directed behaviour, and male behaviour was generally unaffected by female ecotype.

### 3.4. Traits and behaviours of the mating partner affecting aggression

**Figure 6.**
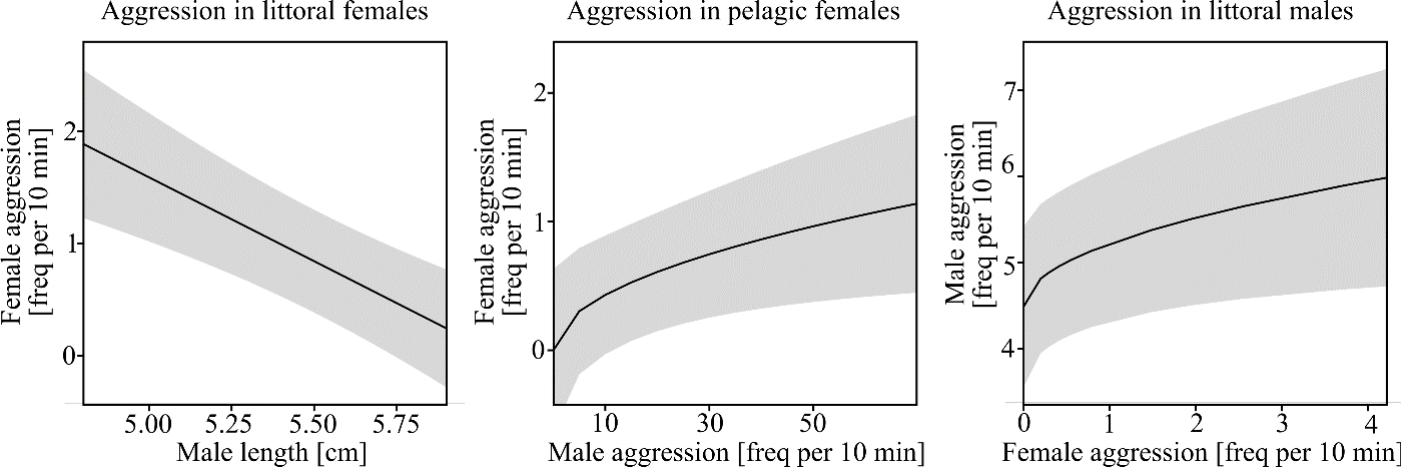
Marginal effect (LMM) of mating partner traits and behaviour on aggression in littoral and pelagic sticklebacks. The shaded grey bands represent 95% confidence intervals.

While aggression in littoral females was best explained by male length (table 3, model 1, *p* < 0.001; figure 6) and male courtship behaviour (table 3, model 1, *p* = 0.019), aggression in pelagic females was best explained by male aggression (table 3, model 1, *p* = 0.035). Aggression in littoral females significantly decreased with increasing male length (*r* = −0.497, *n* = 24, *p* = 0.013) and was positively related to the frequency of male courtship behaviour. Aggression in pelagic females was positively related to male aggression (figure 6). Among female traits and behaviours, fearful behaviour was significantly associated with male aggression, in both littoral males (table 3, model 2, *p* < 0.001) and pelagic males (table 3, model 2, *p* = 0.22). In both male ecotypes, aggression was positively related to female fearful behaviour. Moreover, aggression in littoral males was significantly associated with female aggression (table 3, model 2, *p* = 0.038), with a positive relation between both behaviours. All effect sizes (*η*²) reported in table 3 can be interpreted as large [110].

**Table 3.** Effect of mate characteristics and mate behaviours on the frequency of aggressive behaviour in littoral and pelagic sticklebacks. Effect sizes (*η*²) are presented for all significant terms in the minimum adequate model, which are highlighted in bold font. Numbers in parentheses following the *p*-values indicate the order of removal from the initial model.

mating partner traits and behaviours explaining aggressive behaviour each: d.f. = 1										
	dependent variable	length	Fulton’s condition	aggressive behaviour	agonistic behaviour	courtship behaviour	fearful behaviour	nest-directed behaviour	colouration (hue)	nest quality
model 1	aggressive behaviour in littoral females	***F*_1, 11.1_ = 20.962** ***p* < 0.001** ***η*² = 0.68**	*χ*^2^ = 0.416 *p* = 0.519 (2)	*χ*^2^ = 0.794 *p* = 0.373 (6)	*χ*^2^ = 3.099 *p* = 0.078 (5)	***F*_1, 15.7_ = 6.876** ***p* = 0.019** ***η*² = 0.34**		*χ*^2^ = 0.569 *p* = 0.451 (4)	*χ*^2^ = 0.283 *p* = 0.595 (3)	*χ*^2^ = 0.005 *p* = 0.946 (1)
	aggressive behaviour in pelagic females	*χ*^2^ = 0.094 *p* = 0.759 (2)	*χ*^2^ = 0.650 *p* = 0.420 (4)	***F*_1, 13.7_ = 5.496** ***p* = 0.035** ***η*² = 0.31**	*χ*^2^ = 1.046 *p* = 0.306 (5)	*χ*^2^ = 0.164 *p* = 0.685 (3)		*χ*^2^ = 1.566 *p* = 0.211 (6)	*χ*^2^ = 3.003 *p* = 0.083 (7)	*χ*^2^ = 0.001 *p* = 0.974 (1)
model 2	aggressive behaviour in littoral males	*χ*^2^ = 0.292 *p* = 0.589 (3)	*χ*^2^ = 0.064 *p* = 0.800 (2)	***F*_1, 11.7_ = 5.481** ***p* = 0.038** ***η*² = 0.33**	*χ*^2^ = 2.865 *p* = 0.091 (4)	*χ*^2^ = 0.011 *p* = 0.916 (1)	***F*_1, 13.8_ = 21.299** ***p* < 0.001** ***η*² = 0.63**			
	aggressive behaviour in pelagic males	*χ*^2^ = 0.000 *p* = 0.986 (1)	*χ*^2^ = 0.022 *p* = 0.882 (2)	*χ*^2^ = 1.092 *p* = 0.296 (4)	*χ*^2^ = 0.405 *p* = 0.524 (3)	*χ*^2^ = 1.548 *p* = 0.214 (5)	***F*_1, 21.0_ = 4.854** ***p* = 0.039** ***η*² = 0.22**			

## 4. Discussion

This study gives initial evidence that females of an emerging stickleback ecotype pair exhibit a mating preference for pairings within the same ecotype, although both ecotypes use the same littoral areas within the lake for reproduction. Males of the littoral ecotype turned out to be significantly larger, more aggressive and more intensely coloured than pelagic males and tended to complete nest building more often. Nevertheless, interactions of reproductive females did not primarily depend on these male traits, as females of both ecotypes were significantly more aggressive towards males of the different ecotypes. This finding indicates that females reject males of different ecotypes as mating partners, and the implications of this finding are discussed in the light of an assortative process that might support further adaptation of the two ecotypes to their respective littoral and pelagic habitats.

### 4.1. Phenotypic characterization of the ecotypes

While the reproductively mature littoral and pelagic females used in the experiment did not differ in standard length, weight or condition, differences in these morphometric measurements were apparent in breeding males. Littoral males in the random sample were significantly longer and heavier than pelagic males. There seems to be a general trend for larger body size in the littoral ecotype in Lake Constance, a finding also reported by [72]. A larger body size of the littoral ecotype also characterizes the British Columbian benthic–limnetic species pairs [41–44], suggesting this size difference reflects adaptation to alternative environments, as has been concluded in other studies investigating such variation in stickleback [45–47,50,51,113]. These studies proposed an important role of biomechanical constraints on the feeding apparatus and other environmental factors, such as the spatial distribution of food resources, in shaping stickleback morphology. In Lake Constance, pelagic sticklebacks consume mainly cladocerans and copepods, while littoral sticklebacks typically feed on insect larvae and gammarids [113–116]. Recently [117], it was found that this pelagic prey contains a higher content of polyunsaturated fatty acids that are essential for sticklebacks and rare in the littoral prey items. Exploitation of this high-value food resource might require a small body size in sticklebacks, allowing for a higher foraging efficiency on small prey items [45].

Within sex, neither ecotype differed in the frequencies of courtship, agonistic, fearful and nest-directed behaviours. However, in the case of aggression, the littoral ecotype showed a significantly higher frequency of behaviour than the pelagic ecotype, and this was exclusively observed in males. The level of aggression co-varied with body size and weight, which were both greater in littoral males than in pelagic males. This finding is in concordance with reports from the British Columbian benthic–limnetic species pairs [63], where benthic males court more aggressively [118] and exhibit a greater propensity for destroying the nests of competitors [55]. Furthermore, the British Columbian benthic females engage in nest raids, while such behaviour was never observed in limnetic females [87,119]. One possible explanation for higher aggression in the littoral ecotype might be that this behaviour is beneficial in competition for food resources. The heterogeneous littoral environment may reward competitive behaviour, while lack of macrophytes and structure in the pelagic zone limits the opportunity to monopolize food resources. Thus, aggression may be more adaptive for the littoral ecotype than the pelagic complement.

Another result in our study was that on average, littoral males exhibited a more intense red colouration than pelagic sticklebacks. The red colour is caused by the deposition of carotenoids in the skin of the male throat [120]. Male sex hormones (androgens) mediate the quantity of carotenoid that is diverted to breeding colouration versus somatic maintenance [68,121], while simultaneously facilitating aggressive behaviour [122,123]. There may thus be a causal relationship between the increased aggression and more intense red colouration of littoral males.

### 4.2. Mating preferences

In both ecotypes, the reproductively mature males were building nests and actively courting the females during the experimental trials. The females often responded to these males with a head-up posture, a typical female courtship behaviour that signals spawning readiness [92]. This confirms that the male–female interactions in the experiment reflect a true mating context. It is surprising that only one female laid eggs in the experiment, given that all fish were in full breeding condition, only gravid females were used and the trials were performed right after the peak of the spawning season [124]. This was a pelagic female that spawned in the nest of a pelagic male. Three other females inspected the nest entrance and were close to spawning. Two of them were at the nest of a male of the same ecotype, while only one was at the nest of a male of the different ecotype. The limited space in the aquarium offers a possible explanation for why the females rarely spawned. Males often interfered with the male–female interactions at the other male’s nest as they passed the visual barrier in the middle of the aquarium. Such interference might have disrupted the courtship sequence in many observed interactions and hindered the females from laying eggs.

Males and females courted mating partners of both ecotypes as frequently, and thus courtship behaviour did not specifically indicate a preference. Rejection of mating partners, however, might serve as an indirect indication of preferences. Female aggression can be observed in various contexts, including competition for rank, territory and resources [96,125–127]. Here, female aggression was significantly affected by the interaction of male and female ecotypes, suggesting that females discriminated between male ecotypes and selectively directed more aggression towards the male of different ecotypes. This pattern in female aggression can be interpreted as a rejection of the courtship attempts performed by males of different ecotypes. Aggression has been implied in other vertebrate systems as a mechanism that can lead to reproductive isolation [128–130]. Thus, the divergent aggressive response of sticklebacks found in this study might represent a reproductive barrier.

The reason for stickleback females to reject males of different ecotypes could stem from divergent traits, including differences in body size, male colouration and behaviour. A *post hoc* analysis probing for mate characteristics effects showed that aggression in littoral females was negatively related to male body size. This finding indicates a rejection of the smaller pelagic males. In contrast, male body size had no significant effect on aggression in pelagic females. Overall, this suggests that littoral females discriminate against the significantly smaller pelagic males while pelagic females do not turn away from them. Body size plays an important role in stickleback mate choice. Size assortative mating has been reported in benthic–limnetic [54] and anadromous–freshwater stickleback species pairs [37], where males cease courtship and become aggressive when the size difference between mating partners increases [37,55]. Differences in the behavioural profiles of males of different ecotypes could also contribute to the assortative process, for instance, when pelagic and littoral females begin to respond differently to male behaviour. Specifically, greater aggression in reproductive males might start to discourage courtship and mating in females of the pelagic ecotype but stimulate those behaviours in littoral females [118]. Similarly, a preference for intense male colouration is common in sticklebacks [54], and so variation in sensory biases between females of the two ecotypes might also contribute to an assortative process [61,131]. In the following, we discuss how the observed barriers in mating behaviour could lead to reproductive isolation.

### 4.3. Mechanisms that lead to reproductive isolation

There are several non-exclusive pathways that might, in sequence or simultaneously, lead to reproductive isolation between ecotypes. In theory, these pathways diverge from a common mating system in which females choose males endowed with attractive traits. Across taxa, and especially in fish species that exhibit parental care, male body size is often subject to female sexual selection [132–134]. Females usually prefer larger males, who can be expected to perform better in defence of the eggs [135] and thereby increase recruitment success [136]. Intense breeding colouration is usually also a favoured trait [54,68] since it signals high fitness of the male [121]. The pelagic females in Lake Constance are remarkable in their apparent rejection of littoral males with these attributes, suggesting that ecotype plays a more important role in mate choice than established mating traits. In addition to the visual cues measured in this study, it is possible that females use olfactory cues to recognize males of the same ecotype [66,67]. The ecotype-related behavioural barriers might be complemented by other forms of pre- and postzygotic isolation.

Spatial or temporal separation during reproduction are common facilitators of reproductive isolation [5]. In the lake environment, such separations might arise due to temperature differences in the habitats occupied by the emerging ecotypes. In spring, the pelagic zone heats up more slowly than the littoral [137], and this gradient could result in relatively slower maturation of pelagic stickleback [138]. Hence, reproductive pelagic males and females might arrive later at the spawning sites, which they share with littoral males. Littoral males may thus first occupy the prime nesting sites, which are better vegetated than surrounding areas [139], and they may furthermore be better able to defend these sites since they are more aggressive and larger in size than pelagic competitors [135]. This could lead to micro-partitioning of spawning habitats, as also observed in the British Columbian lakes, where benthic sticklebacks breed in areas with dense vegetation while limnetic sticklebacks spawn on bare sediment [43,63,140]. If females differ in attraction to these microhabitats, this could enhance reproductive isolation, in addition to temporal separation.

## 5. Conclusion

The findings of this study provide the first evidence of emerging behaviour-related reproductive isolation between two ecotypes of stickleback in Lake Constance. Aggressive behaviour was highest in pairings of different ecotypes. Although the underlying reasons for female aggression towards males of different ecotypes remain unknown, we interpret it as rejection. Yet, littoral males are larger, more intensely red-coloured and more aggressive than males of the pelagic ecotype. The divergent aggressive response could feasibly represent an early behavioural differentiation on the spectrum of behaviours that facilitate reproductive isolation of the ecotypes during the early stage of divergence with still weak genetic differentiation.

## Data Availability

All data generated and analysed in this study are available on Figshare [141]. Supplementary material is available online [142].
